# Population Size and Habitat Use by *Tachypleus tridentatus* in the Taiwan Straits

**DOI:** 10.1002/ece3.72332

**Published:** 2025-10-20

**Authors:** Yi‐Jia Shih, Yi‐Hua Jin, Shu‐Chiang Huang, Ya‐Ting Huang, Chia‐Fa Lee, Hernyi Justin Hsieh, Jiun‐Yau Wang, Jia‐Qiao Wang, Yu‐Jia Lin, Chi‐Chin Chen, Yi Chang

**Affiliations:** ^1^ Sustainable Ocean Governance Center National Sun Yat‐sen University Kaohsiung Taiwan; ^2^ Department of Marine Environment and Engineering National Sun Yat‐sen University Kaohsiung Taiwan; ^3^ International Doctoral Program of Marine Science and Technology National Sun Yat‐sen University Kaohsiung Taiwan; ^4^ Graduate Institute of Marine Affairs National Sun Yat‐sen University Kaohsiung Kaohsiung Taiwan; ^5^ Fisheries Research Institute Kinmen County Kinmen Taiwan; ^6^ Penghu Fishery Research Center Fisheries Research Institute, Ministry of Agriculture Penghu Taiwan; ^7^ Marine National Park Headquarters National Parks Service, Ministry of the Interior Kaohsiung Kaohsiung Taiwan; ^8^ Fisheries college Jimei University Xiamen China; ^9^ Marine Science Oriented Ocean Technology Implementation Center National Sun Yat‐sen University Kaohsiung Kaohsiung Taiwan

**Keywords:** climate change, conservation action plan, endangered species, functional habitat, habitat predication

## Abstract

The adult population of *Tachypleus tridentatus* on the main island of Taiwan has long been considered extinct. However, it remains unclear whether this apparent absence reflects true extirpation or simply a lack of sustained large‐scale monitoring. To address this knowledge gap, a multiyear field investigation was conducted from 2018 to 2024 across three key subregions of the Taiwan Strait, namely, Kinmen, Penghu, and mainland Taiwan. Mark–recapture methods were used to estimate the adult population size using two statistical approaches. Population size estimated from the Schnabel method presented 73,629 (95% CI: 59,555–96,415) in Kinmen, 1551 (968–3899) in Penghu, and 1099 (623–4627) in Taiwan main island. Multinomial N‐mixture models showed a population size of 65,428 (63,704–67,156) in Kinmen, 3193 (2818–3588) in Penghu, and 1198 (387–2269) in Taiwan main island. Species distribution modeling (MaxEnt) and connectivity network analyses showed the major habitats in the Taiwan Bank and Changyun Ridge. Recapture records from five tagged individuals supported the predicted dispersal pathways, with the longest recorded straight‐line movement exceeding 110 km, showing ecological connectivity within the Taiwan Strait. Based on these findings, three key conservation strategies are proposed, namely, implementation of standardized long‐term monitoring, habitat restoration and stock enhancement efforts, and preparation for potentially assisted migration under future climate scenarios. The findings have provided a detailed assessment of adult *T. tridentatus* populations in the Taiwan Strait and offer actionable guidance for regional and international conservation planning in the context of environmental changes.

## Graphical abstract

First comprehensive assessment of *Tachypleus tridentatus* population in the Taiwan Strait. Identifies key ecological niches and migration corridors across Taiwan Strait. Proposes a regional conservation strategy for tri‐spine horseshoe crab management.

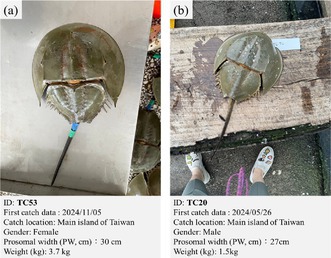

## 1 Introduction

Horseshoe crabs are key indicators with considerable ecological, economic, and cultural implications (Laurie et al. 2019). Their blood is a critical raw material for biomedical testing, especially during the COVID‐19 pandemic, highlighting their commercial value and the need to maintain them as fishery resources (Ashrafuzzaman et al. 2022). The development and survival of horseshoe crabs depend on their access to diverse coastal habitats. Degradation of intertidal zones or increasing marine pollution can adversely affect their growth and recruitment, destabilizing populations and reinforcing their role as coastal ecosystem health indicators. Their morphology and evolutionary lineage have led to their integration into religious rituals and cultural traditions in many coastal communities (Hsieh and Chen 2015; Laurie et al. 2019; Bicknell and Pates 2020). Therefore, the conservation and sustainable use of horseshoe crab populations is critical.

Recently, habitat hotspots for four horseshoe crab species have been successfully predicted using the MaxEnt model (Zhu et al. 2020; Wang et al. 2024; Tang et al. 2024). A summary of the predicted distribution ranges is shown in Table 1. For the three Asian species, the modeled distributions spanned from the Bay of Bengal in western to eastern Indonesia, extending as far north as Japan. Based on these distribution patterns, Wang et al. (2024) delineated seven subregions to examine species‐specific distribution trends. Among Asian taxa, *Tachypleus tridentatus* had the broadest predicted range, covering nearly the entire western Pacific coastline. *Tachypleus gigas* and *Carcinoscorpius rotundicauda* showed substantial habitat overlap. However, the number of predicted hotspots for *C. rotundicauda* was greater than that for *T. gigas* . All three Asian species are restricted to regions west of Wallace's line. In contrast, *Limulus polyphemus* is geographically isolated from its Asian counterparts and has a broad distribution along the Atlantic coast of North and Central America (Zhu et al. 2020).

**TABLE 1:** Potential distribution ranges of the four extant horseshoe crab species predicted by Maxent models, classified by biogeographic subregions.

Species	*Tachypleus tridentatus* ^a^	*Tachypleus gigas* ^a^	*Carcinoscorpius rotundicauda* ^a^	*Limulus polyphemus* ^b^
Potential distribution based on the Maxent models	The most widespread potential distribution range. East Asia subregion. North Southeast Asia subregion. South Southeast Asia subregion. South Asia subregion. Philippines. Sulawesi (Indonesia).	The narrowest among the three Asian species. East Asia subregion. North Southeast Asia subregion. South Southeast Asia subregion. South Asia subregion.	East Asia subregion. North Southeast Asia subregion. South Southeast Asia subregion. South Asia subregion.	Gulf of Maine. Mid‐Atlantic. Southeast. Florida Atlantic. Northeast Gulf of Mexico. Yucatán Peninsula.

^a^ The definition of subregions for Asian horseshoe crabs follows Wang et al. (2024).

^b^ While the delineation of six metapopulations for American horseshoe crabs is based on Zhu et al. (2020).

Both *T. tridentatus* and *L. polyphemus* exhibit wide geographic ranges, and the spatial predictions generated by these models offer critical insights for transboundary conservation planning and strategic design of marine protected areas. However, *L. polyphemus* benefits from well‐established management frameworks that contribute to long‐term population stability (Smith et al. 2023). However, *T. tridentatus* continues to face severe threats from habitat degradation, environmental changes, anthropogenic pressure, and overfishing (Chen et al. 2004; Hsieh and Chen 2015; Kwan et al. 2018; Wang et al. 2024). Consequently, the International Union for the Conservation of Nature (IUCN) classified *T. tridentatus* as Endangered in 2019, highlighting the urgent need to strengthen conservation efforts in East Asia (Laurie et al. 2019; Wang et al. 2024; Chen et al. 2024).

The effective conservation of *T. tridentatus* requires data on its population dynamics and particularly baseline information on its distribution and population size. This is essential for developing sustainable management strategies and ensuring effective species conservation. This is particularly true for species such as horseshoe crabs, which occupy distinct habitats at different life stages (Laurie et al. 2019; Chen et al. 2024). Juveniles typically inhabit the intertidal zones, whereas adults migrate to shallow offshore waters (Laurie et al. 2019). However, to date, prior research has primarily focused on juveniles in intertidal zones, addressing topics such as their distribution, abundance, habitat degradation, and artificial stock enhancement (Vestbo et al. 2018; Chen et al. 2004; Hsieh and Chen 2015; Kwan et al. 2018; Jin et al. 2025).

In contrast, studies on adult *T. tridentatus* remain limited because of sampling challenges. This has resulted in critical gaps in our understanding of adult population size, migratory behavior, and reproduction (Chen et al. 2024; Wang et al. 2024). Understanding adult *T. tridentatus* dynamics is essential for evaluating range expansion, habitat use, and long‐term population viability. A sufficient number of sexually mature adults must successfully return to their spawning sites and lay viable eggs to ensure adequate recruitment and sustain population growth (Ricker 1954; Krebs 2016; Manca et al. 2017; Chen et al. 2024; Wang et al. 2024). Given that adults are far more mobile than juveniles, their distribution patterns and habitat connectivity must be understood to ensure that functional habitat networks support their reproductive success and genetic diversity (Almendral and Schoppe 2005; Manca et al. 2017; Laurie et al. 2019; Chen et al. 2024; Wang et al. 2024). Wang et al. (2024) further emphasized that establishing long‐term baseline data on adult distributions is critical for validating species distribution models and providing a scientific foundation for implementing effective conservation measures. These include habitat protection and management, sustainable resource use, stock enhancement planning, ex situ conservation efforts, and promoting regionally coordinated strategies (Vestbo et al. 2018; Laurie et al. 2019; Wang et al. 2024).

*T. tridentatus* abundance has been examined in Malaysia, Japan, and the South China Sea using various approaches, including mark–recapture, manual counting during spawning events, and density interpolation (Wada et al. 2010, 2016; Manca et al. 2017; Laurie et al. 2019; Chen et al. 2024). However, these methods introduce different bias types that hinder direct comparisons across regions. Manual counting is typically limited to accessible spawning beaches and may underestimate populations in less‐surveyed areas or during nonpeak breeding periods. Density interpolation often relies on sparse or opportunistic sampling, potentially leading to an overestimation in data‐poor regions. Among these, the mark–recapture method is more statistically robust for estimating population size. However, it is labor‐intensive and sensitive to variations in recapture probabilities caused by environmental conditions or individual behavior (Ricker 1954; Swan 2005; Krebs 2016; Manca et al. 2017). Despite these limitations, mark–recapture remains the most widely accepted method because of its ability to incorporate detection probabilities and generate reliable population estimates (Ricker 1954; Swan 2005; Krebs 2016; Manca et al. 2017).

MaxEnt modeling has led to substantial advances in predicting the distribution of *T. tridentatus* , particularly in Asia and the Beibu Gulf region of China, and has provided various conservation planning proposals (Chen et al. 2024; Wang et al. 2024). However, most studies in this context have primarily focused on identifying potential habitats. Analyses addressing how this broadly and continuously distributed species uses functional habitats, and how spatial connectivity is maintained among core areas, remain limited. Bridging these knowledge gaps will provide valuable insights into the development of more effective and ecologically grounded conservation strategies for *T. tridentatus* (Hilty et al. 2020; Delina et al. 2023; Wang et al. 2023).

Although the Taiwan Strait has been consistently identified as a suitable habitat for *T. tridentatus* (Chen et al. 2004; Hsieh and Chen 2015; Kwan et al. 2018; Wang et al. 2020, 2024), Hsieh and Chen (2015) reported a rapid population decline in Taiwan. This suggests that the species might have undergone regional extinction on Taiwan's main island. This assessment, lacking subsequent empirical updates, has been widely cited and incorporated into the IUCN 2019 regional extinction evaluation.

Historical records indicate that the Taiwan Strait, including Kinmen, Penghu, and the Taiwan main island, supported active populations of *T. tridentatus* during the 1950s and the 1960s (Chen et al. 2004; Hsieh and Chen 2015; Laurie et al. 2019). As a major oceanic corridor linking the South China Sea and East China Sea, the Taiwan Strait plays a critical ecological role (Delina et al. 2023; Wang et al. 2023). Recent genetic analyses by Tang et al. (2024) showed a north–south population structure separating the Southeast Asian and Japanese lineages, implying both a substantial dispersal capacity and the potential role of the Taiwan Strait as a biogeographic connector. Given this context, the absence of recent records near the Taiwan main island raises an important question: Has the local population truly been extirpated, or has its apparent absence resulted from a lack of long‐term, large‐scale monitoring?

**FIGURE 1:**
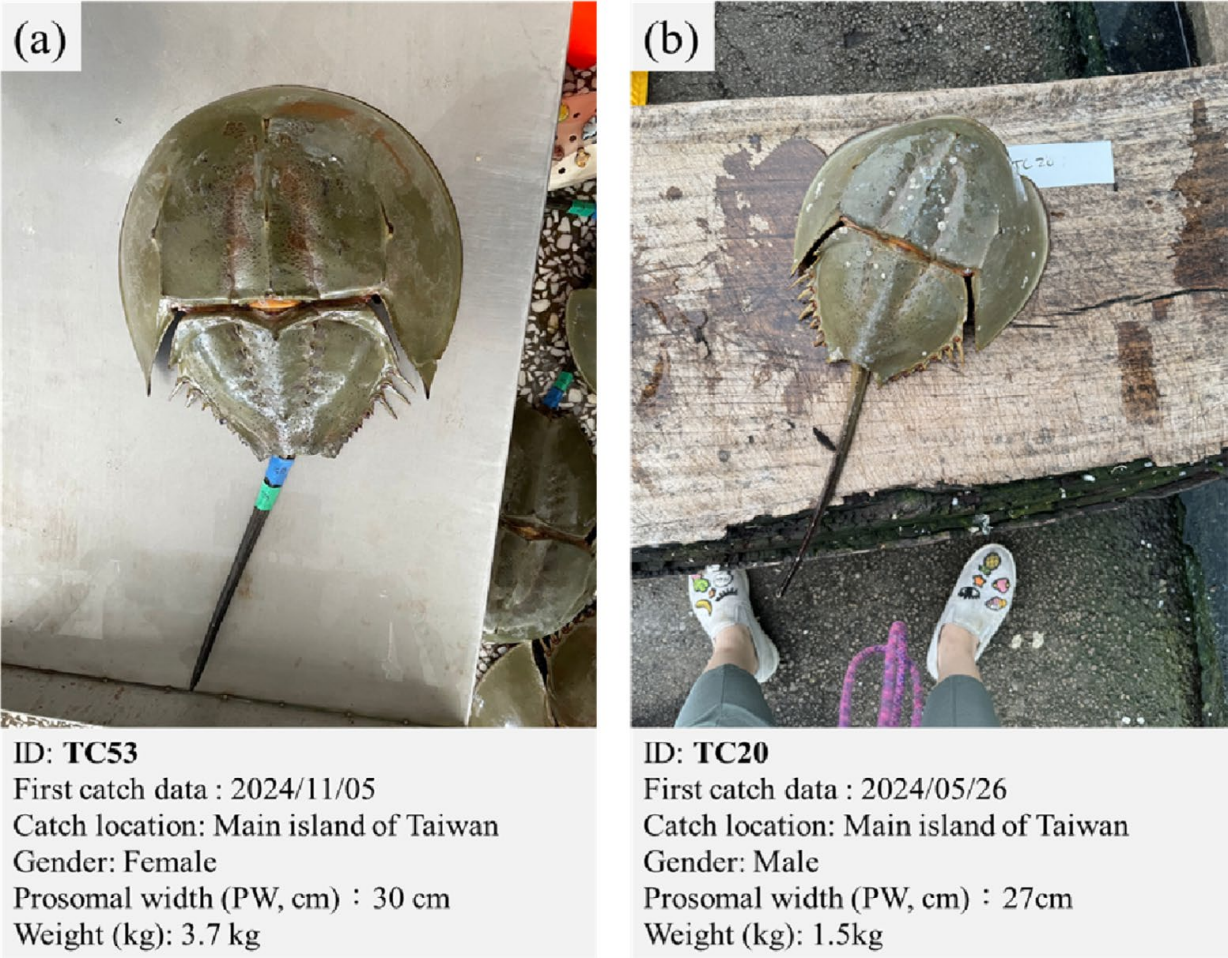
Adult *Tachypleus tridentatus* individuals collected from the coastal waters off Taiwan main island. (a) Female (ID: TC53) and (b) male (ID: TC20). Both specimens were tagged and released as part of a long‐term monitoring program. Photographs were taken by the authors.

Therefore, in this study, we aimed to conduct systematic field investigations across three key subregions, Kinmen, Penghu, and the Taiwan main island of the Taiwan Strait. Here, historical records document the presence of adult *T. tridentatus* (Figure 1). By integrating mark–recapture methods with species distribution modeling (MaxEnt) and connectivity network analysis using ArcGIS Pro, we aimed to identify population hotspots, infer potential migratory corridors, and assess the habitat service functions of the Taiwan Strait. These findings can contribute critical baseline data to support future conservation and management strategies for *T. tridentatus* .

## 2 Materials and Methods

### 2.1 Study Period, Specimen Collation, and Fishing Effort Estimation

**FIGURE 2:**
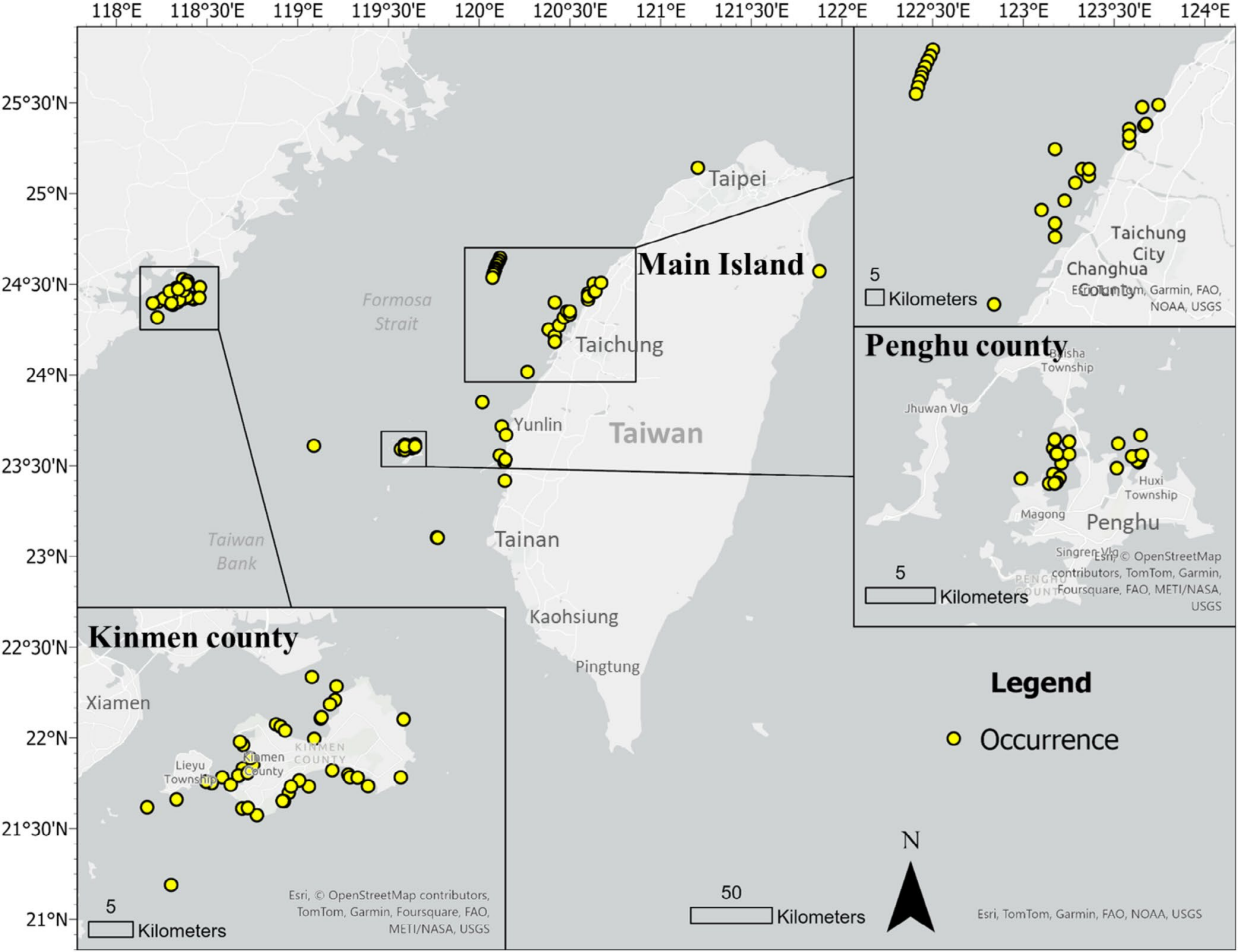
Sampling locations and specimen abundance during the study period.

The study area covered three subregions of Taiwan, namely, Kinmen County, Penghu County, and Taiwan's main island (Figure 2). Due to differences in local regulations and operational practices, sampling methods varied across the three study areas and are described as follows: (1) *Kinmen*: sample collection in Kinmen was performed from 2018 to 2024, except for 2019, when sampling was suspended due to COVID‐19. In 1999, Kinmen established Taiwan's first *T. tridentatus* conservation zone in Guningtou. Since then, continuous local government outreach and early involvement by the Kinmen Fisheries Research Institute in captive breeding programs have encouraged fishermen to report or surrender captured individuals in exchange for financial incentives. From 2018 to 2021, only basic biological data on adult horseshoe crabs were recorded, with no detailed information on fishing efforts. Starting in 2022, the catch location and number of individuals were systematically recorded. Given the diverse harvesting methods used in Kinmen, including hand collection and the use of gillnets in the intertidal zone, with variable deployment durations, standardizing gear‐based effort was not feasible. Therefore, fishing effort was quantified based on the number of individuals captured per fisher per fishing event (individuals/per person time). (2) *Penghu*: Specimens were collected during two survey periods: March to October 2023 (8 months) and April to October 2024 (7 months). Field sampling was conducted through permitted operations using chartered fishing vessels in accordance with the fishing bans issued under the Fisheries Act. The fishing gear used was bottom‐set gillnets with a mesh size of 3.5 in., a net height of 150 cm, and a length of approximately 100 m. Nets were deployed on sand–muddy substrates at depths of 3–5 m nearshore and were retrieved after 24 h. The fishing effort was standardized and recorded as the number of individuals per net set (individuals/net). (3) *Taiwan Main Island*: sampling began in October 2023 and continued until December 2024. With over 200 fishing ports along the coast, samples were collected in collaboration with the Taiwan Fishery Sustainability Development Foundation, which manages the landing declarations for coastal and nearshore fisheries across major ports. During routine operations, if a horseshoe crab was reported as a bycatch, the team was contacted. Specimens were then purchased based on the prevailing auction prices at each port. Catch records included vessel names, fishing locations, and gear types. Most specimens were collected using gillnets, trawls, and nets. Preliminary data suggest that most individuals were caught in nearshore waters, although a few vessels operated further offshore. Owing to the variability in gear types and fishing duration, fishing effort was also calculated as the number of individuals per person per fishing trip (individuals/per person time).

### 2.2 Target and Tracking

**FIGURE 3:**
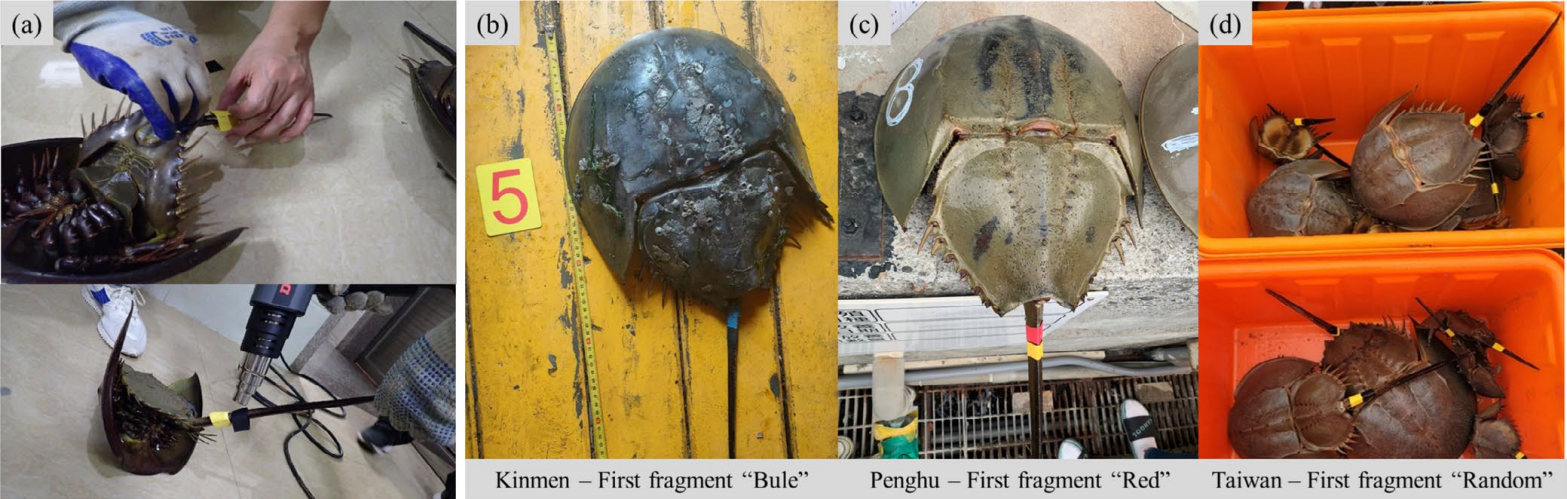
Markers and targeted processing for each location and individual. (a) Processing of marked individuals, (b) first fragment color in Kinmen County, (c) first fragment color in Penghu County, and (d) first fragment color in Taiwan.

Commercially available colored heat‐shrinking tubing (Taiwan Intelligent Sensor Technology Co. Ltd.; Product No. TOL‐004423; material: polyolefin; shrink ratio: 2:1; operating temperature: −55°C to 125°C) was used for individual identification. Tubing with internal diameters ranging from 10 to 16 Ø, depending on body size, was cut into 5 cm fragments. Each fragment was marked with a unique identification number using a permanent marker and then heated to 60°C using a hot air gun to secure it at the base of the tail (Figure 3a). Two colors were used per individual to indicate both the subregional origin and release year (Figure 3b). The first color represents the subregion of origin: Kinmen, blue; Penghu, red; and Taiwan Main Island, random (Figure 3b–d). The second color, that is, red, yellow, green, white, or black, indicates the release year for each tagged individual. In Kinmen, where a long‐term tagging program has been sustained for over 7 years, a different second color was assigned each year to differentiate the release cohorts. During the initial phase of the study, individuals were tagged without written identification numbers (ID). The color‐coding scheme used in Kinmen was as follows: red for 2018, yellow for 2020, green for 2021, white for 2022, black for 2023, and red with an additional ID number for 2024.

When the study commenced in each subregion, a few marked individuals were temporarily held in aquaculture ponds corresponding to their capture sites (Kinmen and Penghu: Fisheries Research Institute; Taiwan Main Island: National Sun Yat‐sen University). These individuals were maintained for approximately 1 week to 1 month following tagging to ensure that the marking procedure did not adversely affect their health or survival. After preliminary testing, all the marked individuals were released immediately at or near their original capture sites.

### 2.3 Estimate Population Size Model I—Schnabel Method

The population of horseshoe crabs was assumed to follow a closed model, with individuals undergoing multiple capture–recapture events. The Schnabel method based on Krebs (2016) and Manca et al. (2017) was used for population estimates. The formula used is as follows:

$$\hat{N}=\frac{\sum_{t}\left(\;C_{t}M_{t}\right)}{\sum_{t}R_{t}+1}$$

$$\text{Variance}\;\left\{\frac{1}{\hat{N}}\right\}=\frac{\sum R_{t}}{\sum {\left(C_{t}M_{t}\right)}^{2}}$$

$$\mathrm{SE}\;\text{of}\;\frac{1}{\hat{N}}=\sqrt{\frac{\sum R_{t}}{\sum {\left(C_{t}M_{t}\right)}^{2}}}$$

where *N*: population estimate; C_*t*_: total number of individuals caught in sample *t*; M_*t*_: number of marked individuals in the population just before the *t*; R_*t*_: number of individuals already marked when caught in sample *t*.

After calculating the estimates and variances for each subregion, a Bayesian framework was used to compare the differences among the three subregions. This approach offers a flexible means of estimating credible intervals and assessing differences without assuming normality or equal variance. All the analyses were conducted using Python 3.12.6 (released in September 2024).

### 2.4 Estimate Population Size Model II—Multinomial N‐Mixture Models

All the capture–recapture analyses were conducted in a Python 3.10 environment managed through the Anaconda distribution (https://www.anaconda.com/). We applied a multinomial N‐mixture model (Royle 2004; Dorazio et al. 2005) in a Bayesian setting using the PyMC v5 probabilistic programming framework (Mollenhauer and Brewer 2017). This model was conceptually equivalent to that used by the unmarked R package (Kellner et al. 2023), and the site‐specific abundance was assumed to follow a log‐normal prior distribution, and detection probability (*p*) was assumed to follow a Beta distribution.

Posterior inference was performed using the No‐U‐turn Sampler (NUTS), a variant of the Hamiltonian Monte Carlo, with four parallel chains, 2000 posterior draws, and 1000 tuning steps. We used a target acceptance rate of 0.95 to stabilize sampling. The model convergence and effective sample size were evaluated using the Arviz Diagnostic Toolkit (https://www.arviz.org/). Graphical outputs and data summaries were generated using Matplotlib, Pandas, and Seaborns.

### 2.5 Predicting Suitable Habitat and Pathways in Taiwan Strait

#### 2.5.1 Species Distribution and Environmental Data Preparation

Empirical observational data from adult horseshoe crabs were collected. Local fishers carefully documented each specimen, including its latitude and longitude. These locations provided baseline information on the habitats of the species. Selecting and processing environmental variables followed the method established in previous habitat modeling studies of *T. tridentatus* . This emphasizes benthic marine factors as key determinants of their spatial distribution and seasonal dynamics (Chen et al. 2024; Wang et al. 2024). The rasters of seven sea benthic factors were used at 5 km spatial resolution, including temperature (SBT, °C), salinity (SBS), primary productivity (SB‐PP, mmol/m^3^), dissolved oxygen (SB‐DO, mmol/m^3^), water velocity (SB‐WV, m/s), bathymetry (Bathy, m), and topographic slope (Slo, °) were downloaded as the average depth and annual mean data of present layers from Bio‐ORACLE, v3.0. The layers were standardized to the spatial resolution at 1 km through the function “Resample” using bilinear interpolation, with an output cell size of 0.01 in ArcGIS Pro 3.3.1 (Esri). The rasters were then converted to ASCII format for the MaxEnt modeling using the “Raster to ASCII” function.

#### 2.5.2 Species Distribution Modeling

We conducted SDM using the MaxEnt software (version 3.4.4, available at https://biodiversityinformatics.amnh.org/open_source/maxent/). The MaxEnt model, which is based on the principle of maximum entropy, establishes a relationship between species occurrence records and various environmental conditions to predict species distribution or assess habitat suitability (Elith et al. 2011). The parameter settings for MaxEnt were adapted from those outlined by Wang et al. (2024): (1) selected “Do jackknife to measure variable importance”; (2) used the default output format “Cloglog” for presence probability estimation; (3) selected “random seed”; (4) set the “random test percentage” to 25; (5) specified “replicates” as 50; (6) set “replicated run type” to subsample; and (7) established “maximum iterations” at 5000 for convergence. All other settings were set to the default values.

The receiver operating characteristic curve method was used to calculate the area under the curve (AUC) to evaluate the model's performance (Phillips et al. 2006; Elith et al. 2011). The jackknife analysis tool was used to assess the contribution of each environmental variable and determine the impact of each variable on the distribution of *T. tridentatus* . The Cloglog model output yields probability values ranging from 0 to 1 (Phillips et al. 2017). These are proportional to species occurrence and are defined as the habitat quality index (HQI). Here, higher index values indicate greater suitability for species survival (Chen et al. 2024).

#### 2.5.3 Connectivity Network Analysis

Constructing a connectivity network for a specific species requires identifying key habitats within the landscape that warrant protection (dispersal sources) and understanding the environmental factors influencing species dispersal (Wang et al. 2024; Chen et al. 2024). Such information was generated from the species distribution modeling outputs and used to create the core area and ecological resistance surface for the connectivity network analysis. We conducted a connectivity network analysis in ArcGIS Pro using the Linkage Pathways Tool from the Linkage Mapper Toolbox (version 3.1.0, available at https://linkagemapper.org/) to construct a marine connectivity network for *T. tridentatus* in the study area. The linkage pathway tool uses ArcGIS and Python scripts to identify neighboring core areas and generate maps of the least‐cost corridors between them (McRae and Kavanagh 2011). We reclassified species distribution modeling areas from the MaxEnt model output by dividing the HQI values into quartiles and defining those in the fourth quartile (Q4, 75%–100%) as the core area, the critical zone needing protection.

## 3 Results

### 3.1 Population Structure of *Tachypleus tridentatus* in Three Taiwan Strait Subregions

Between 2018 and 2024, 5707 catch times of adult *T. tridentatus* were recorded across Taiwan. Among them, 3898 individuals were tagged with 167 recaptures, resulting in a recapture rate of approximately 4.28% (167/3898). In Kinmen, the 6‐year average recapture rate was 4.02%, with values ranging from 3.60% to 6.70% across the years, except for an anomalously low rate in 2022 (0.77%). In Penghu, the recapture rate over the 2 years was 6.10%, while in Taiwan, the average was 7.21% (Table 2).

**TABLE 2:** Estimated adult population size (value in bold font) of *Tachypleus tridentatus* in Taiwan. Model I as Schnabel method (in green); Model II as multinomial N‐mixture models (in orange).

Model	Population estimates of years	Subregions									
		Kinmen						Penghu		Taiwan main island	
		2018	2020	2021	2022	2023	2024	2023	2024	2023	2024
Total number of individuals captured		134	91	343	919	1300	2216	133	146	12	141
Marker individuals		128	86	332	910	862	1239	90	124	12	115
Recaptured individuals		5	4	15	7	31	83	3	11	1	7
Recaptured rate		3.73%	4.40%	4.37%	0.76%	2.38%	3.75%	2.26%	7.53%	8.33%	4.96%
I	**Population size (*n*)**	**1888**	**3313**	**7968**	**107,623**	**67,541**	**73,629**	**1121**	**1551**	**72**	**1099**
	Lower 95% CI	1,041	1,770	5,217	62,549	48,973	59,555	577	968	17	623
	Upper 95% CI	10,046	25,798	16,862	385,246	108,784	96,415	19,849	3899	549	4627
II	**Population size (*n*)**	**16,356**	**21,809**	**65,427**	**54,524**	**65,425**	**65,428**	**2394**	**3193**	**115**	**1198**
	Lower 95% CI	15,929	21,232	63,687	53,051	63,698	63,704	2108	2818	23	387
	Upper 95% CI	16,784	22,371	67,179	55,947	67,178	67,156	2681	3588	235	2269

The capture efforts across the three subregions are summarized in Table 3. The annual catch‐per‐unit effort (CPUE) in Kinmen between 2022 and 2024 ranged from 4.30 to 4.60 individuals/person time. In Taiwan, the CPUE ranged from 2.27 to 3.00 individuals/person time during 2023 to 2024. In Penghu, the CPUE was calculated based on net units, with values between 3.67 and 4.21 individuals/net from 2023 to 2024. Detailed monthly CPUE dynamics are shown in Table S1.

**TABLE 3:** Capture efforts (catch‐per‐unit effort, CPUE) across the three subregions, while CPUE definition (in orange) in Penghu was different from the other two regions.

	Subregions						
	Kinmen (individual/per person time)			Taiwan (individual/per person time)		Penghu (individual/per net)	
	2022	2023	2024	2023	2024	2023	2024
Total person times or total net	259	302	511	4	62	36	63
Catch effort (CPUE)	4.60	4.30	4.34	3.00	2.27	3.67	4.21

The distribution of prosomal width across the three subregions is shown in Figure S1. The prosomal widths of the collected individuals ranged from approximately 10 to 40 cm during the study period. Female specimens reached a maximum prosomal width of 40 cm, which was primarily observed in samples from the main island of Taiwan. Meanwhile, the smallest females that were approximately 10 cm were mainly found in Penghu (Figure S1a). Male individuals reached up to 34 cm, with large males recorded both on the Taiwan Main Island and in Kinmen. The smallest males (~9 cm) were also observed in Penghu (Figure S1b). The size–frequency distribution of the captured individuals suggests that these tagged individuals are likely to have at least one more year of life expectancy.

Population abundance was estimated using two different models, and the results are summarized in Table 2. For Model I—Schnabel method, Kinmen had the longest time series among all the subregions. The estimates were relatively low from 2018 to 2021, followed by a pronounced peak in 2022. Since 2023, both capture and recapture numbers have increased substantially, and estimates have stabilized accordingly. In Penghu, consistent capture and recapture efforts yielded relatively stable population estimates for both years. In Taiwan, tagging and monitoring began only in October 2023, resulting in a low population estimate for that year (72 individuals, CI: 17–549). The estimate for 2024 increased to 1099 individuals (CI: 623–4627).

**FIGURE 4:**
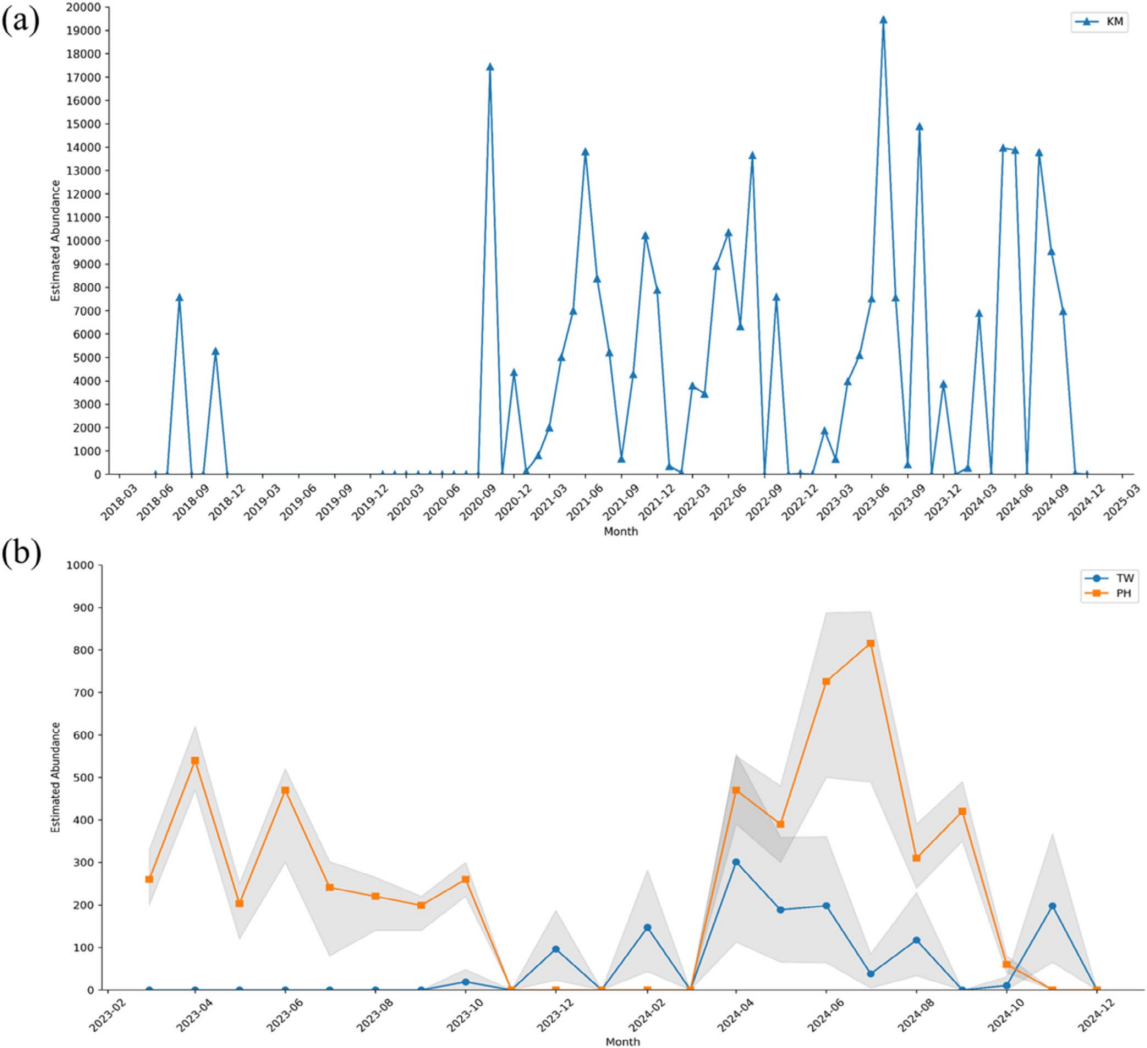
Monthly estimates of *Tachypleus tridentatus* abundance across three subregions in the Taiwan Strait based on Multinomial N‐mixture Models. (a) Estimated monthly abundance in Kinmen (KM) from 2018 to 2024 (except 2019) showing variable population size with temporal clustering. (b) Estimated monthly abundance in Penghu (PH, orange) and the Taiwan main island (TW, blue) from 2023 to 2024. Shaded areas indicate 95% confidence intervals. The model outputs suggest seasonal fluctuations in apparent abundance, with distinct peaks observed during spring to summer months.

For Model II, Multinomial N‐mixture models, annual estimates, and associated confidence intervals for each subregion are presented in Table 2, with the monthly estimates detailed in Table S2. In Kinmen, the long‐term data improved the detection probability at each sampling occasion. The estimated population in 2018 was 16,356 individuals (CI: 15,929–16,784). As observations accumulated over time, the estimated population size increased, reaching 65,428 individuals by 2024 (CI: 63,704–67,156). Monthly dynamics (Figure 4a) showed that adult horseshoe crab abundance began to increase annually in March, peaking between June and August. The highest estimated monthly abundance was observed in 2023 at 19,465 individuals (CI: 19,457–19,474). In Penghu, the population was estimated to be approximately 3000 individuals per year across the 2 years. Monthly dynamics (Figure 4b, orange line) showed a peak abundance in April and June 2023 and a gradual increase beginning in April 2024, peaking in July. The July 2024 estimate was the highest recorded over the 2‐year period, with 816 individuals (CI: 489–890). In Taiwan, the estimated population size was 115 individuals (CI: 23–235) in 2023, increasing to 1198 individuals (CI: 387–2269) in 2024. The monthly dynamics (Figure 4b, blue line) showed the highest estimate in April 2024 for 301 individuals (CI: 112–554). 197 individuals (CI: 64–367) were identified in November 2024, indicating continued habitat use in late autumn.

Regardless of the model applied, the results consistently indicated that the Kinmen subregion had the largest population of *T. tridentatus* . Meanwhile, Taiwan supported the smallest population among the three subregions. The monthly dynamics across all subregions indicated increased use of nearshore habitats by adult *T. tridentatus* during the spring and summer months. When the observation time was limited, the traditional Schnabel method tended to yield more conservative estimates. This highlights that this method is more reliant on long‐term data monitoring.

### 3.2 Predicting Suitable Distribution Areas

**FIGURE 5:**
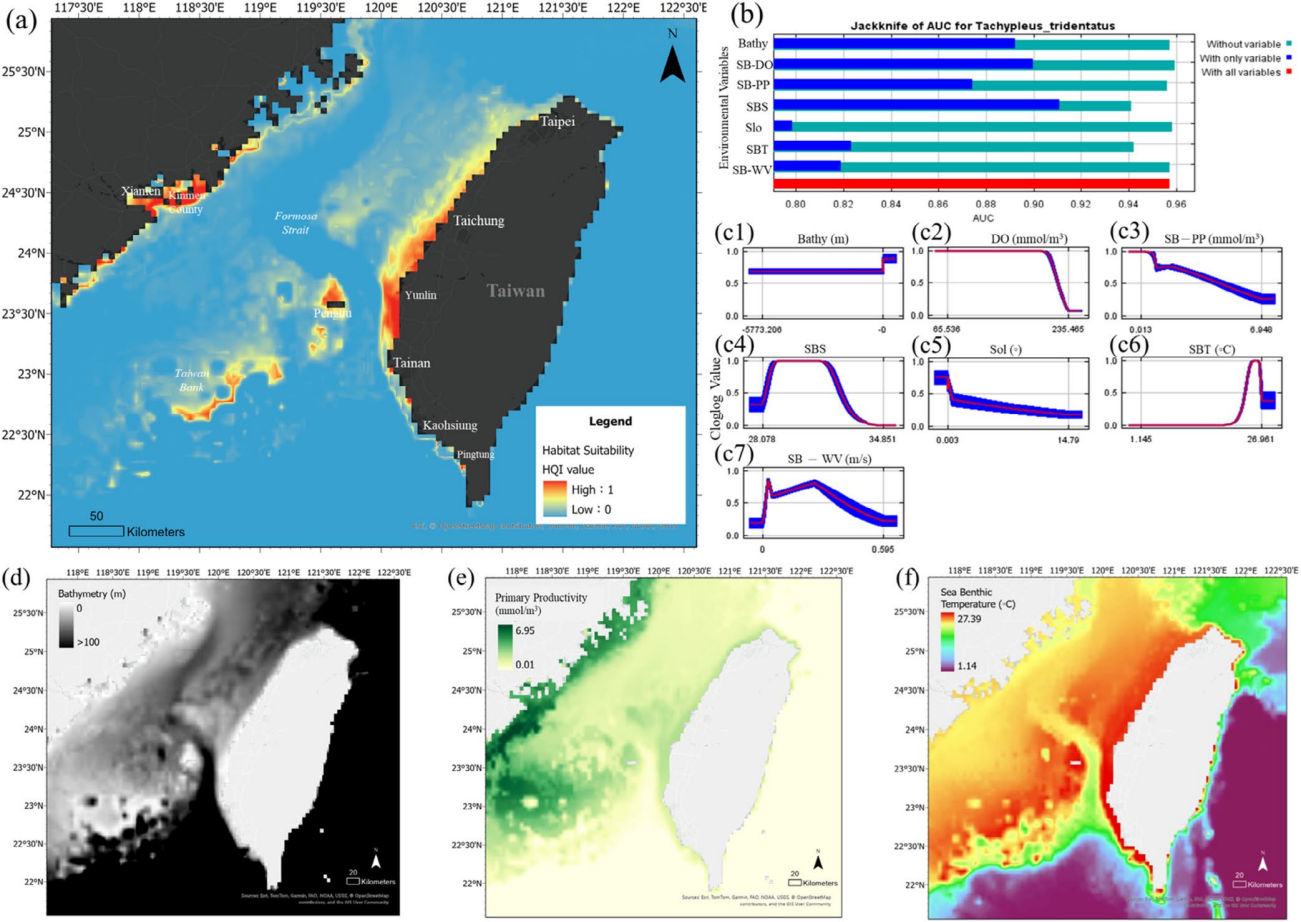
Predicted distribution model and core region analysis for *Tachypleus tridentatus* . (a) Maxent model prediction map showing the potential habitat hotspots for *T. tridentatus* across the Taiwan Strait and adjacent waters. Dark colors indicate higher habitat suitability index (HQI) values, with particularly high suitability observed along the southwestern coast of Taiwan and the southeastern coast of Fujian, China. (b) Jackknife test of variable importance based on AUC values. Bathymetry and sea benthic dissolved oxygen (SB‐DO) contributed the most to model performance, indicating their primary roles in determining the species' distribution. (c1–c7) Response curves for the seven environmental variables influencing HQI values. Variables: (c1) bathymetry, (c2) SB‐DO, (c3) sea benthic primary productivity (SB‐PP), (c4) sea benthic salinity (SBS), (c5) topographic slope (Slo), (c6) sea benthic temperature (SBT), and (c7) sea benthic water velocity (SB‐WV). Each curve represents the mean response of habitat suitability to the respective variable (blue line) with standard deviation (shaded area) showing *T. tridentatus* ecological niche preference. (d) Average bathymetry (m); (e) annual average sea benthic primary productivity (mmol/m^3^); (f) annual average sea benthic temperature (°C).

The locations of the collected species are shown in Figure 2. A total of 48 core habitat areas were predicted based on the observed latitudes and longitudes of the distribution data. Eight of these were located in the central channel of the Taiwan Strait, far from the coastline. These predicted habitats for the adult population were not randomly distributed across the Taiwan Strait but were concentrated in both the inshore and central strait areas. The locations of inshore habitats have been supported by long‐term research and have mainly contributed to the breeding and nursing functions of juvenile horseshoe crabs. The central Strait areas included the Taiwan Bank and the Changyun Ridge, where a high probability of occurrence was predicted (Figure 5a). The results show that potentially functional habitats may have long been excluded from prior surveys.

The jackknife test of variable contribution (Figure 5b) indicated that bathymetry (percentage contribution with SD: 40.13 ± 5.20), primary productivity (19.07 ± 3.13), and temperature (16.17 ± 2.60) were the top three influential predictors for the *T. tridentatus* distribution (Table 4). The ecological preferences associated with each predictor variable (Figure 5c1–c7) were further characterized. Bathymetry (Figure 5c1,d) was the most influential factor, with the highest contribution to habitat suitability. In the Taiwan Strait, the average annual bathymetry is approximately 50 m, and the highest habitat suitability is associated with shallow waters ranging from 0 to 20 m. Sea benthic primary productivity (Figure 5c3,e) showed a suitability peak within the range of 0.01 to 6.95 mmol/m^3^, with an annual average of approximately 2.06 mmol/m^3^. This indicates a preference for moderately productive benthic environments in the Taiwan Strait. Sea benthic temperature (Figure 5c6,f) showed optimal suitability in the range of 20°C–27°C. The average annual temperature in the Taiwan Strait was around 22°C–23°C, and deviations outside this temperature range corresponded to decreased habitat suitability. These findings suggest that *T. tridentatus* favors shallow coastal areas with moderate primary productivity and stable temperate benthic conditions.

**TABLE 4:** Percentage contributions (mean ± standard deviation) of environmental predictors to the habitat suitability model for *Tachypleus tridentatus* in Taiwan Strait.

Predictor	Percentage contribution (mean ± standard deviation)
Bathymetry (m)	**40.13 ± 5.20**
Dissolved oxygen (mmol/m^3^)	6.25 ± 0.83
Primary productivity (mmol/m^3^)	**19.07 ± 3.13**
Salinity (PSS)	14.05 ± 1.45
Slope (°)	1.73 ± 0.64
Temperature (°C)	**16.17 ± 2.60**
Water velocity (m/s)	2.60 ± 0.60

*Note:* Bathymetry, primary productivity (Chlorophyll a concentration), and temperature were identified as the top three influential variables that given in bold font.

### 3.3 Connectivity Networks in the Taiwan Strait

**FIGURE 6:**
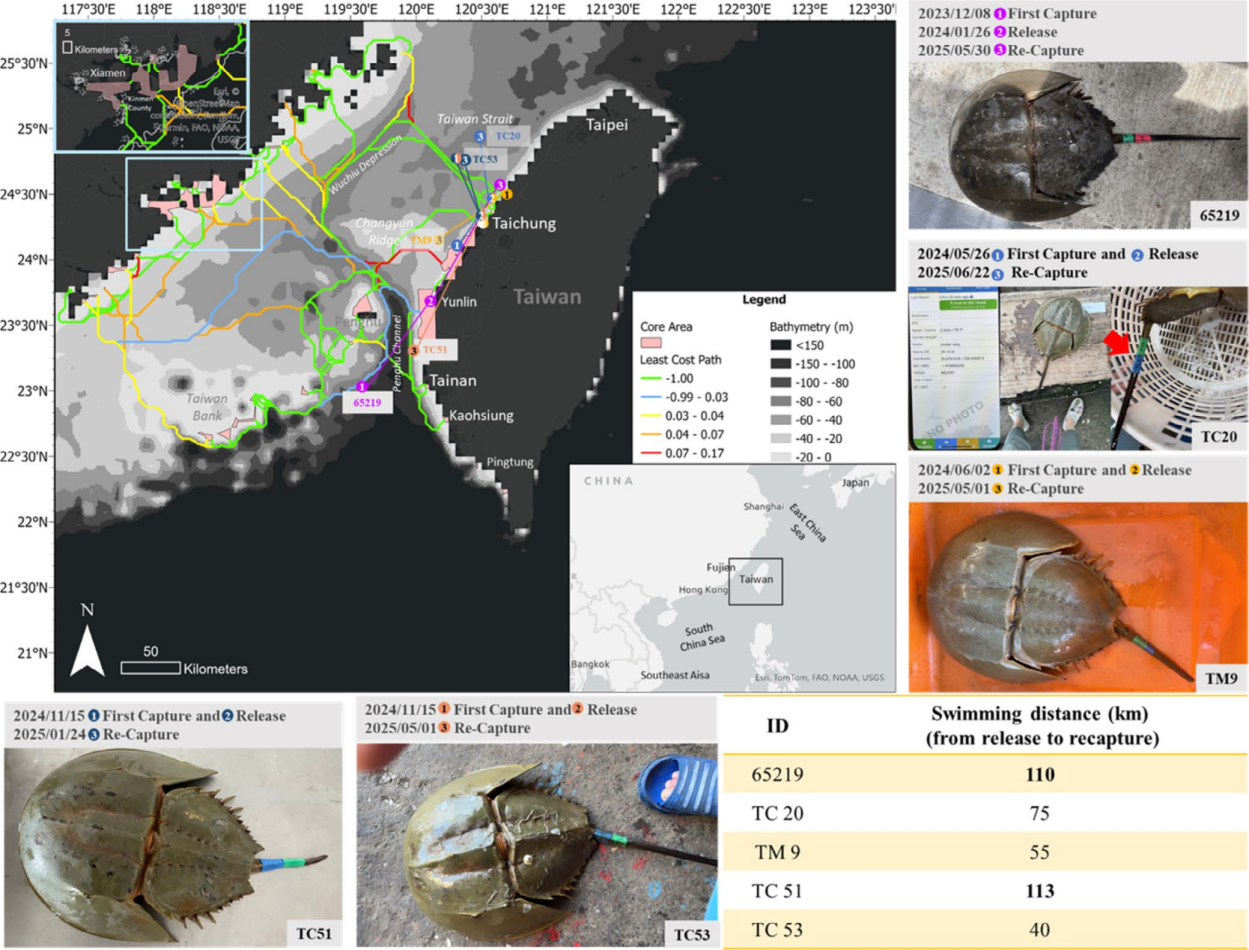
Connectivity network mapping of *Tachypleus tridentatus* in the Taiwan Strait region, integrating modeled least‐cost pathways and empirical mark–recapture data. The background map depicts predicted least‐cost paths (color gradients) overlaid on bathymetric layers, with core habitat areas outlined. Colored lines represent potential dispersal routes between sampling sites. Overlaid photographs show representative tagged individuals and their corresponding recapture locations, with dates of first capture/release and recapture annotated. Estimated straight‐line movement distances (in kilometers) between release and recapture sites are shown in yellow panels. These integrated results validate the model predictions and show potential adult dispersal connectivity among habitats surrounding the Taiwan main island.

Based on the 48 core regions, the connectivity network was mapped and 138 linkages across the Taiwan Strait were identified based on the lowest cost path values (Figure 6). Green lines represent high‐probability corridors chosen by *T. tridentatus* . Meanwhile, red lines indicate low‐probability corridors. All linkages were classified into three types: (1) along the inshore, these linkages were predicted to be along the coastlines on each side of the strait, and those pathways were shown as high use; (2) in the middle of the Taiwan Strait, these pathways were mainly located in the Taiwan Bank–Penghu Archipelago area. Here, *T. tridentatus* is predicted to move through the central strait, also represented as high use; and (3) across the Taiwan Strait: the results showed several potential cross‐strait corridors. The main corridors include Dongshan Island, Fujian–Taiwan Bank, Pingtan, Fujian–Wuchiu Depression–Penghu Channel–Taiwan inshore, and Quanzhou, Fujian–Penghu Channel–Taiwan inshore. These pathways range in depth from 60 to 80 m and extend over 350 km. The cross‐strait corridors, primarily represented by yellow, blue, and green lines, indicated moderate to high use, suggesting strong interactions between *T. tridentatus* populations on both sides of the strait.

As evidence, several individuals tagged on Taiwan's main island in 2024 were recaptured. Individual 65,219 (Figure 6, purple dot), one of the first adult *T. tridentatus* strains tagged by our team, was recaptured after 490 days. This individual was initially caught on December 8, 2023, in the offshore waters of Penghu (23.103200° N, 119.772400° E). After temporary rearing for 2 months at the National Sun Yat‐sen University to observe the effects of tagging, it was released on January 26, 2024, at 23.639917° N, 120.142389° E. This individual was later recaptured on May 30, 2025, off the coast of Miaoli County (24.506174° N, 120.673341° E). This confirmed that the tag remained attached and that the individual survived for over 1 year in the wild. The distance between the release and recapture sites was approximately 110 km.

Another male individual, TC20, was first reported on May 26, 2024, when it was captured using an inshore bottom‐set gillnet near Taichung (24.183333° N, 120.416667° E; Figure 6, light blue dot). It was tagged and released on the same day on a nearby beach in the catch area (24.437580° N, 120.617859° E). On June 20, 2025, it was recaptured using a trawler at 25.075140° N, 120.426050° E, approximately 75 km from the release site. Twenty‐three previously unrecorded individuals were captured at this location.

Individual TM9 (Figure 6, orange dots) was caught and tagged using a gillnet on June 2, 2024, at 24.461943° N, 120.641439° E, and released on the same day in nearby Miaoli County (24.451035° N, 120.638953° E). It was recaptured 333 days later, on May 1, 2025, at 24.161170° N, 120.198140° E, with a straight‐line distance of approximately 55 km. Two additional individuals, TC51 (Figure 6, dark blue dot) and TC53 (Figure 6, dark orange dot), were captured by offshore trawling on November 5, 2024, near Taichung Port (24.721369° N, 120.383821° E), and released near Lishui Fishing Port (24.201512° N, 120.490851° E). TC51 was recaptured on January 24, 2025, off the coast of Qigu, Tainan City (23.289322° N, 119.962448° E), with a straight‐line movement of approximately 113 km. TC53 was recaptured on May 1, 2025, by gillnets fishing off Taichung (24.449744° N, 120.200379° E), approximately 40 km from its release site. The integration of the predicted migration pathways with actual recapture data provides valuable insights into the connectivity network of the Taiwan Strait.

## 4 Discussion

### 4.1 Population Status of Adult *Tachypleus tridentatus* in Taiwan

Assessing adult population dynamics is essential to guide conservation policies and evaluate their effectiveness (Ricker 1954; Almendral and Schoppe 2005; Krebs 2016). In this study, we used the traditional mark–recapture method, in which data credibility and accuracy were verified through recapture rates and variance, which formed the key reliability indicators (Pine et al. 2003; Rigby et al. 2012; Grimm et al. 2014). First, for the recapture rate, the recapture rate was the lowest (1.5%) in Malaysia, whereas *L. polyphemus* accounted for 7% in a 17‐year study (Swan 2005). In this study, the overall recapture rate was 4.28%, ranging from 3.60% to 7.21% across the three subregions. Although the differences in these indicators may be due to species‐specific population dynamics, habitats that promote high mobility, or biases from long‐term data collection (Pine et al. 2003; Rigby et al. 2012; Grimm et al. 2014), the stable recapture rate and low variance observed further support the benefits of long‐term research in improving the accuracy of population estimates and reducing bias (Pine et al. 2003; Swan 2005; Rigby et al. 2012; Grimm et al. 2014; Krebs 2016). Owing to operational and regulatory constraints in each subregion, the sampling strategy was not randomized. Consequently, certain locations may have been over‐ or under‐represented. Although the CPUE was calculated to help standardize comparisons, the specific characteristics of the Penghu subregion still introduce limitations. These constraints highlight the need for more standardized and systematic sampling designs in future research.

Cross‐year recaptures were frequently observed in this study. One female released into the Kinmen waters in 2018 was recaptured 6 years later, in May 2024, in the southwestern sea of Kinmen, with a body weight of 1850 g at recapture. This individual had the longest tracking duration observed in the study. However, as no ID numbers were assigned during the early phase of the tagging program, the exact month of release in 2018 could not be determined. Carapace length frequency analysis indicated that most captured individuals retained their molting potential. Therefore, a natural mortality rate of zero was assumed for population size estimation in this study.

Further comparisons of *T. tridentatus* adult populations across Asia showed that recent reports are available only for Malaysia, Beibu Gulf, China, and Japan (Wada et al. 2010, 2016; Laurie et al. 2019; Chen et al. 2024). While Taiwan and Malaysia used consistent estimation methods, Japan relied on direct counts, and Beibu Gulf population abundance was estimated by calculating catch density (ind./km^2^) based on the specific swept area of the otter trawl. Of these regions, only Taiwan and Malaysia showed comparable baseline values. Otherwise, the results also highlighted the Taiwan Strait as one of the few regions reporting a substantial adult population in recent years, emphasizing the potential role of the ecological niche in the Taiwan Strait. The findings emphasize the necessity of integrating long‐term mark–recapture studies into management strategies and indicate the critical importance of standardized methods for enabling effective cross‐regional comparisons and evaluating conservation effectiveness.

### 4.2 The Taiwan Strait as a Predicted Migration Pathway for Adult *Tachypleus tridentatus*

Understanding the spatial distribution of animals is essential for assessing habitat health, connectivity, and species conservation of marine organisms (Shuter et al. 2011; Hilty et al. 2020; Teng et al. 2021; Chen et al. 2024; Wang et al. 2024). Recently, predictive models for species distribution and movement have advanced substantially (Hilty et al. 2020; Teng et al. 2021; Delina et al. 2023; Laurie et al. 2019; Chen et al. 2024). Among these, the MaxEnt model has become one of the most widely used tools for habitat suitability modeling, owing to its high spatial resolution, strong environmental interpretability, and robust performance with presence‐only data (Merow et al. 2013; Wang et al. 2024). However, a key limitation of the traditional MaxEnt framework is its reliance on static environmental predictors, typically long‐term annual or multidecadal averages. These may fail to reflect short‐term climatic variability or seasonal extremes associated with species occurrence records, thereby constraining its capacity to model dynamic ecological processes, such as seasonality shifts in species distributions (Barthel et al. 2025). The MaxEnt model was selected based on several key considerations. First, our dataset did not include temporally matched environmental variables. Therefore, long‐term average environmental layers were used. Second, MaxEnt offers strong ecological interpretability through outputs, such as variable contribution scores and response curves. These provide detailed insights into the environmental factors influencing habitat suitability. These features are particularly valuable for formulating conservation plans and guiding habitat restoration. Third, we aimed to maintain method consistency and ensure comparability with previous studies on *T. tridentatus* (Wang et al. 2024; Chen et al. 2024), most of which have used the MaxEnt approach. Although we acknowledge the growing use of ensembles and other machine learning methods, MaxEnt remains a well‐established and transparent modeling framework, especially suited for presence‐only data and data‐limited scenarios. Therefore, this method framework was used in this study. In future studies, we plan to incorporate real‐time environmental variables and examine the applicability of alternative modeling approaches.

Collecting adult *T. tridentatus* specimens remains challenging, with most studies focusing on juvenile crabs in intertidal zones (Chen et al. 2004, 2024; Wang et al. 2024; Jin et al. 2025). Although recent studies have provided valuable predictions of adult population distributions, only Chen et al. (2024) incorporated direct capture data to identify over 100 ecological corridors and key adult habitats in Beibu Gulf. In contrast, the predictions of Wang et al. (2024) and Tang et al. (2024) were extrapolated to the Taiwan Strait based on ecological and distributional data obtained from other geographic regions. However, Wang et al. (2024) avoided discussing habitat suitability for Taiwan's main island because of its designation as a regionally extinct area for *T. tridentatus* (Chen et al. 2004; Hsieh and Chen 2015). However, Zhu et al. (2020) emphasized that when niche divergence exists among populations, the distribution of one group cannot be reliably used to model habitat suitability for another. This highlights the importance of region‐specific habitat suitability assessments, particularly for areas such as the Taiwan main island, which has been repeatedly identified in past modeling efforts as a potentially suitable but unverified habitat zone. The selection of the MaxEnt model for this study was based on several key considerations. First, MaxEnt provides strong ecological interpretability through outputs, such as variable contribution scores and response curves. These offer detailed insights into the environmental conditions associated with species habitat suitability. These features make them particularly useful in informing conservation actions and guiding habitat restoration. Second, we aimed to maintain methodological consistency and comparability with previous studies on *T. tridentatus* (Wang et al. 2024; Chen et al. 2024), most of which adopted the MaxEnt approach.

*T. tridentatus* has traditionally been regarded as a predominantly coastal species with limited migratory capacity (Wada et al. 2016; Jin et al. 2025). Therefore, the connectivity of its migratory pathways has received limited attention. However, Tang et al. (2024) recently demonstrated that this species is genetically structured into two distinct populations separated by the East China Sea. This indicates a greater dispersal capability across broad geographic distances than previously assumed. The Taiwan Strait is a critical biogeographic corridor linking the East and South China Seas. This type of narrow coastal passage supports essential functions for marine organisms, including feeding, breeding, and shelter corridors (Shanks 2020; Lowerre‐Barbieri et al. 2021). Therefore, by integrating previous molecular evidence with our recapture data, we have addressed a key knowledge gap by providing direct empirical support for the hypothesis that habitat patches within the Taiwan Strait are ecologically connected and facilitate the active movement of adult *T. tridentatus* .

These results suggest that the Taiwan Strait offers several known or potential ecological functions for *T. tridentatus* , including (1) *Spawning grounds*: and that the increased use of coastal areas during the spawning season of adult horseshoe crabs highlights the importance of these habitats (Laurie et al. 2019; Chen et al. 2024; Jin et al. 2025). The coastal corridors along both sides of Xiamen Bay have a higher likelihood of usage and stronger connectivity, reinforcing the role of these areas in reproduction. (2) *Migration and activity pathways*: Benthic invertebrates such as mollusks, crustaceans, and echinoderms often use ocean currents to enhance their dispersal (Briscoe et al. 2021; Hsiao et al. 2021; Secor et al. 2024; Wang et al. 2024). By riding the current, these organisms can travel long distances with minimal energy expenditure (Chang et al. 2010; Lee et al. 2015; Secor et al. 2024). Our study has demonstrated that most cross‐strait migratory routes of *T. tridentatus* pass along the channel edges of the Changyun Ridge. Along this route, the flow direction and velocity show seasonal variations (Jan et al. 2006; Lee et al. 2015; Hsieh et al. 2023), that could play a key role in facilitating the movement of adult populations between core habitats in the Taiwan Strait. However, empirical evidence from this study supports the predicted movement and connectivity patterns across the Taiwan Strait. Further long‐term research is needed to validate these patterns and enhance our understanding of the underlying ecological and behavioral dynamics. (3) *Potential foraging grounds*: During the nonbreeding season, adult horseshoe crabs typically migrate away from coastal areas and descend into semi‐enclosed bay environments with flat, sandy, or muddy bottoms. These offer shelter and abundant food resources (Laurie et al. 2019). In this study, we have identified the Taiwan Bank, off southern Taiwan in the Taiwan Strait, as a potential foraging ground for adult horseshoe crabs due to nutrient inflows and a sandy substrate that supports high biodiversity (Jan et al. 2006; Hsiao et al. 2021; Hsieh et al. 2023; Huang et al. 2024).

Juvenile *T. tridentatus* was not included in our movement modeling. Upon hatching from buried eggs, juveniles settle rapidly on benthic substrates and exhibit limited dispersal capacity during their early developmental stages (Chen et al. 2004). Therefore, our analysis focused exclusively on adult individuals, who are more likely to engage in long‐distance movements across coastal habitats.

The movement corridors identified in this study were constructed by combining habitat suitability outputs from MaxEnt with a least‐cost path analysis based on environmental resistance surfaces. This approach is commonly used in ecological connectivity studies to predict potential dispersal pathways under realistic environmental conditions. Although the method is predictive, its reliability was supported by recently obtained recapture data. These have provided empirical evidence of long‐distance movements by adult individuals across the Taiwan Strait.

Current tagging systems use external visual markers without tracking or telemetry capabilities. Movement distances were estimated using straight‐line measurements between the release and recapture points. While these findings offer preliminary support for the species' dispersal potential, they do not allow for the reconstruction of detailed movement paths or transitions between specific habitat types.

Although a few individuals were captured in deep offshore waters south of Taiwan, we re‐evaluated and excluded these deep‐water areas from our connectivity model. This adjustment was made to ensure that the final interpretation of adult movement potential remained conservative and consistent with both environmental suitability and the available empirical evidence.

### 4.3 Regional Conservation Network in Taiwan Strait

In China, the *T. tridentatus* has been listed in the second category of portaged species, meaning that fishing harvests have been prohibited on the Fujian coast since 2021 (National Forestry and Grassland Administration and Ministry of Agriculture and Rural Affairs of China 2021). In Taiwan, conservation measures for *T. tridentatus* in Kinmen County were established by establishing the Guningtou Horseshoe Crab Conservation Zone in 1999, based on the Fisheries Act. This prohibits the harvest of *T. tridentatus* in 2025. Meanwhile, Penghu and Lienchiang Counties prohibited the harvest of *T. tridentatus* in their authority water 3 nm from the coast from 2021 (Huang et al. 2024). However, conservation measures in Taiwan are lacking, and inconsistent regulations between China and Taiwan may lead to low conservation efficiency. Local or nationwide conservation actions do not reduce the extinction risk of the entire population (Wang et al. 2024). Therefore, we have suggested specific measures for the regional conservation networks of *T. tridentatus* and other widely migrating species. The following three directions outline feasible and implementable conservation measures: (1) *Standardized long‐term monitoring as a foundation for population assessment*: Data from the three subregions examined in this study show that long‐term monitoring substantially reduces uncertainty in population size estimates, regardless of the statistical model applied. Within the broader Indo‐Pacific region, only Taiwan and Malaysia currently offer comparable baseline data (Manca et al. 2017). This highlights the urgent need for standardized monitoring protocols and transnational data‐sharing platforms to support the effective assessment and conservation of regional populations. (2) *Habitat restoration and population enhancement*: the integration of least‐cost path analysis, empirical tagging data, and previous genetic studies suggests that populations within the Taiwan Strait likely represent a single, interconnected population unit. Based on this, we recommend two complementary strategies to support population recovery: (a) *Habitat protection and restoration*. Coastal habitats in both Taiwan and Fujian have experienced degradation due to urban development and shoreline modification. Jin et al. 2025 documented habitat loss in Xiamen Bay, highlighting the urgency of implementing conservation measures for the remaining natural coastlines and planning restoration efforts in ecologically suitable areas. (b) *Stock enhancement*: Artificial propagation and juvenile release are widely used strategies in Taiwan and China to support population recovery (Xu et al. 2021). However, key limitations remain, including the need to optimize the release size, maintain genetic diversity, and conduct long‐term survival monitoring. Our future research will focus on population genetics and genetic variability on both sides of the Taiwan Strait to inform sustainable and scientifically grounded stock enhancement programs. (3) *Preparation for assisted migration under climate change*: Our results emphasize that the Taiwan Strait functions as a connected habitat supporting the movement, foraging, and potential reproduction of *T. tridentatus* , with adult individuals capable of dispersing across subregional boundaries. It has been projected that climate‐driven changes in habitat suitability will lead to the loss of equatorial habitats and a potential range expansion to higher latitudes (Tang et al. 2024). The results of species distribution modeling and environmental preference analyses (Figure 5) confirmed that *T. tridentatus* prefers shallow waters, moderate primary productivity, and temperate benthic conditions, which are abundant in the Taiwan Strait (Figure 5d–f). Given its role as a potential climate refugee and dispersal hub, international cooperation is urgently required to safeguard the existing habitats (Dawson et al. 2011; Bonebrake et al. 2018). Such actions will ensure that the Taiwan Strait continues to provide essential ecological functions for the resident and migrating populations under future climate scenarios.

## 5 Conclusions

In this study, we assessed the adult *T. tridentatus* population and its habitat connectivity in the Taiwan Strait over decades, bridging substantial gaps in baseline data. The population in the Taiwan Strait is conservatively estimated to exceed 60,000 individuals per year. The data also emphasize the critical role of long‐term monitoring and detailed sampling in achieving reliable population estimates.

Habitat prediction and recapture evidence showed that adult *T. tridentatus* habitats are concentrated in both the inshore and central strait regions. Central areas such as the Taiwan Bank and Changyun Ridge have been overlooked for a long time. Connectivity analysis identified 138 potential migration pathways, emphasizing the ecological importance of the Taiwan Strait as a key breeding ground, migratory corridor, and foraging habitat. These findings highlight the role of the Taiwan Strait in connecting the populations of Taiwan and the eastern coast of China.

Based on the ecological importance of the Taiwan Strait for *T. tridentatus* , regional conservation strategies have been proposed and cross‐national collaboration has been emphasized. Recommendations include the establishment of unified conservation measures, periodic reviews of protected areas, international data‐sharing platforms, and continuous monitoring to assess conservation outcomes. This study lays the groundwork for developing a holistic conservation framework that contributes to the sustainability of *T. tridentatus* populations and their habitats across the Taiwan Strait.

## Author Contributions

**Yi‐Jia Shih:** conceptualization (lead), data curation (lead), formal analysis (lead), investigation (lead), methodology (lead), project administration (equal), resources (equal), software (supporting), validation (supporting), visualization (equal), writing – original draft (lead), writing – review and editing (equal). **Yi‐Hua Jin:** data curation (supporting), formal analysis (supporting), investigation (lead), methodology (supporting), software (supporting), writing – original draft (supporting). **Shu‐Chiang Huang:** data curation (supporting), formal analysis (supporting), methodology (supporting), software (supporting), visualization (supporting), writing – original draft (supporting). **Ya‐Ting Huang:** data curation (supporting), investigation (lead), resources (supporting), software (supporting), visualization (supporting). **Chia‐Fa Lee:** data curation (supporting), investigation (supporting), resources (supporting). **Hernyi Justin Hsieh:** data curation (supporting), investigation (supporting), resources (supporting). **Jiun‐Yau Wang:** data curation (supporting), investigation (supporting), resources (supporting). **Jia‐Qiao Wang:** data curation (supporting), investigation (supporting), resources (supporting). **Yu‐Jia Lin:** methodology (supporting), software (equal), writing – original draft (supporting). **Chi‐Chin Chen:** investigation (supporting), resources (supporting). **Yi Chang:** conceptualization (lead), data curation (lead), formal analysis (lead), funding acquisition (lead), investigation (supporting), methodology (lead), project administration (lead), resources (lead), supervision (lead), visualization (supporting), writing – original draft (supporting), writing – review and editing (lead).

## Disclosure

*Declaration of Generative AI and* *AI‐Assisted Technologies in the Writing Process*: During the preparation of this study, the author(s) used ChatGPT 4o to improve language. After using this tool/service, the author(s) reviewed and edited the content as needed and took full responsibility for the content of the publication.

## Conflicts of Interest

The authors declare no conflicts of interest.

## Supporting information


**Figure S1:** Distribution of prosomal width across the three subregions. (a) Female; (b) male.
**Table S1:** Capture efforts (catch‐per‐unit effort, CPUE) across the three subregions in monthly. The period of Kinmen was between 2022 and 2024; Penghu and Taiwan main island was between 2023 and 2024.
**Table S2:** Monthly estimates population across three subregions based on Multinomial N‐mixture Models.

**Appendix S1:** ece372332‐sup‐0002‐AppendixS2.xlsx.

## Data Availability

The raw data generated and analyzed in this study are publicly available at the following link: https://docs.google.com/spreadsheets/d/1a_WchEfCOLdfp_t7BC1PY0T6NMp1WCk7/edit?usp=drive_link&ouid=115743795245135950610&rtpof=true&SD=true.
