# Supplementary material for: Population Size and Habitat Use by Tachypleus tridentatus in the Taiwan Straits
Source: Ecol Evol. 2025 Oct 20;15(10):e72332. doi: 10.1002/ece3.72332 (PMC12537299; doi:10.1002/ece3.72332)
Supplement: Supplementary file 1 — Figure S1: Distribution of prosomal width across the three subregions. (a) Female; (b) male. Table S1: Capture efforts (catch‐per‐unit effort, CPUE) across the three subregions in monthly. The period of Kinmen was between 2022 and 2024; Penghu and Taiwan main island was between 2023 and 2024. Table S2: Monthly estimates population across three subregions based on Multinomial N‐mixture Models. [file ECE3-15-e72332-s001.docx]

**
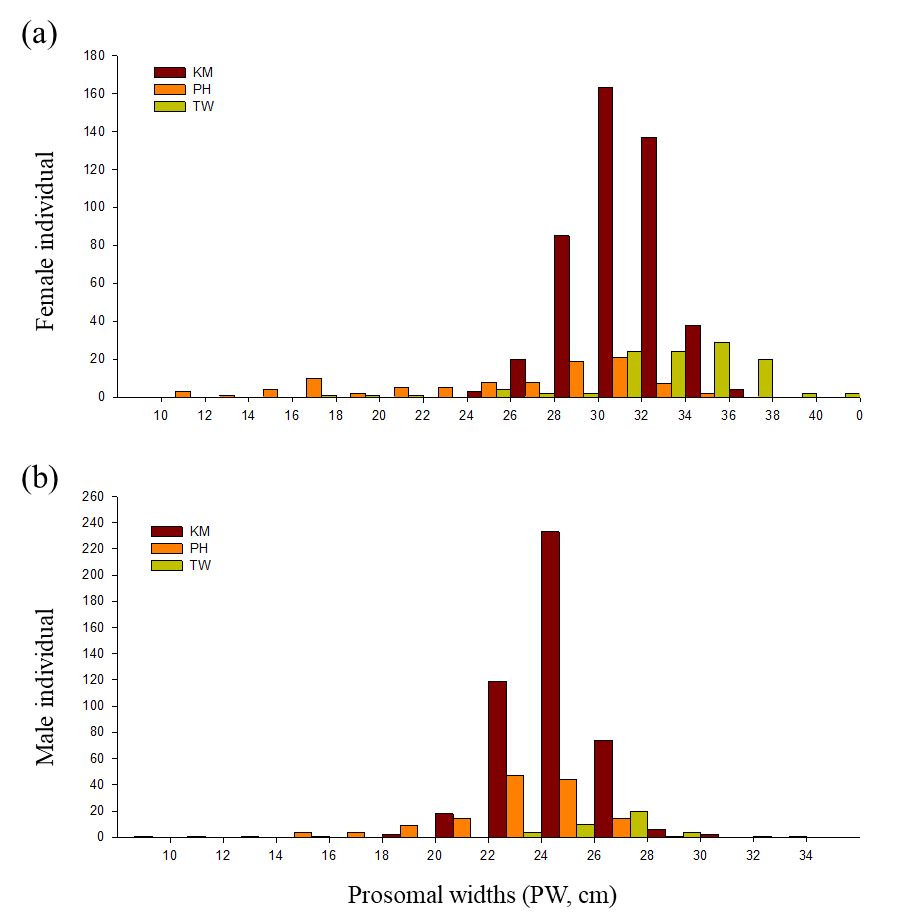
**

**Fig. S1** The distribution of prosomal width across the three subregions. (a) Female; (b) Male.

**Table S1** Capture efforts (Catch per unit effort, CPUE) across the three subregions in monthly. The period of Kinmen was between 2022 to 2024; Penghu and Taiwan main island was between 2023 to 2024.

| Subregion | | Kinmen | | | Penghu | | | Taiwan (Main island) | | |
| --- | --- | --- | --- | --- | --- | --- | --- | --- | --- | --- |
| Year | Month | individual | per person | CPUE | individual | per person | CPUE | individual | per person | CPUE |
| 2022 | Jan | 9 | 5 | 1.80 | - | - | - | - | - | - |
| 2022 | Feb | 2 | 1 | 2.00 | - | - | - | - | - | - |
| 2022 | Mar | 28 | 11 | 2.55 | - | - | - | - | - | - |
| 2022 | Apr | 122 | 40 | 3.05 | - | - | - | - | - | - |
| 2022 | May | 292 | 67 | 4.36 | - | - | - | - | - | - |
| 2022 | Jun | 263 | 63 | 4.17 | - | - | - | - | - | - |
| 2022 | Jul | 432 | 62 | 6.97 | - | - | - | - | - | - |
| 2022 | Aug | 15 | 8 | 1.88 | - | - | - | - | - | - |
| 2022 | Oct | 25 | 8 | 3.13 | - | - | - | - | - | - |
| 2022 | Dec | 3 | 2 | 1.50 | - | - | - | - | - | - |
| 2023 | Jan | - | - | - | - | - | - | - | - | - |
| 2023 | Feb | 4 | 2 | 2.00 | - | - | - | - | - | - |
| 2023 | Mar | 28 | 16 | 1.75 | 17 | 4 | 4.25 | - | - | - |
| 2023 | Apr | 94 | 29 | 3.24 | 26 | 5 | 5.20 | - | - | - |
| 2023 | May | 112 | 26 | 4.31 | 12 | 4 | 3.00 | - | - | - |
| 2023 | Jun | 268 | 47 | 5.70 | 20 | 5 | 4.00 | - | - | - |
| 2023 | Jul | 418 | 71 | 5.89 | 7 | 3 | 2.33 | - | - | - |
| 2023 | Aug | 198 | 39 | 5.08 | 12 | 5 | 2.40 | - | - | - |
| 2023 | Sep | 11 | 4 | 2.75 | 20 | 4 | 5.00 | - | - | - |
| 2023 | Oct | 67 | 30 | 2.23 | 18 | 6 | 3.00 | 2 | 1 | 2.00 |
| 2023 | Nov | - | - | - | - | - | - |  |  |  |
| 2023 | Dec | 100 | 36 | 2.69 | - | - | - | 10 | 3 | 3.33 |
| 2024 | Jan | - | - | - | - | - | - | - | - | - |
| 2024 | Feb | 6 | 3 | 2.00 | - | - | - | 15 | 3 | 5.00 |
| 2024 | Mar | 148 | 14 | 2.71 | - | - | - | - | - | - |
| 2024 | Apr | - | - | - | 16 | 5 | 3.20 | 35 | 17 | 2.06 |
| 2024 | May | 301 | 94 | 4.78 | 14 | 5 | 2.80 | 24 | 16 | 1.50 |
| 2024 | Jun | 433 | 110 | 3.94 | 29 | 6 | 4.83 | 26 | 15 | 1.73 |
| 2024 | Jul | - | - | - | 38 | 6 | 6.33 | 6 | 3 | 2.00 |
| 2024 | Aug | 420 | 84 | 5.00 | 10 | 2 | 5.00 | 13 | 3 | 4.33 |
| 2024 | Sep | 568 | 100 | 5.68 | 24 | 2 | 12.00 | - | - | - |
| 2024 | Oct | 154 | 46 | 2.48 | 2 | 1 | 2.00 | 2 | 2 | 1.00 |
| 2024 | Nov | 188 | 13 | 2.85 | - | - | - | 20 | 3 | 6.67 |

**Table S2** Monthly estimates population across three subregions based on Multinomial N-mixture Models.

| Subregion | | Kinmen | | | Penghu | | | Taiwan (Mainland) | | |
| --- | --- | --- | --- | --- | --- | --- | --- | --- | --- | --- |
| Year | Month | Population size (n) | Lower 95% CI | Upper 95% CL | Population size (n) | Lower 95% CI | Upper 95% CL | Population size (n) | Lower 95% CI | Upper 95% CL |
| 2018 | May | 3,480 | 3,476 | 3,484 | - | - | - | - | - | - |
| 2018 | Aug | 7,575 | 7,570 | 7,580 | - | - | - | - | - | - |
| 2018 | Nov | 5,280 | 5,276 | 5,284 | - | - | - | - | - | - |
| 2020 | Oct | 17,447 | 17,438 | 17,456 | - | - | - | - | - | - |
| 2020 | Dec | 4,361 | 4,352 | 4,370 | - | - | - | - | - | - |
| 2021 | Jan | 134 | 132 | 136 | - | - | - | - | - | - |
| 2021 | Feb | 802 | 800 | 804 | - | - | - | - | - | - |
| 2021 | Mar | 2,005 | 2,001 | 2,009 | - | - | - | - | - | - |
| 2021 | Apr | 5,013 | 5,009 | 5,017 | - | - | - | - | - | - |
| 2021 | May | 7,001 | 6,994 | 7,008 | - | - | - | - | - | - |
| 2021 | Jum | 13,816 | 13,809 | 13,823 | - | - | - | - | - | - |
| 2021 | Jul | 8,381 | 8,374 | 8,388 | - | - | - | - | - | - |
| 2021 | Aug | 5,214 | 5,209 | 5,218 | - | - | - | - | - | - |
| 2021 | Sep | 668 | 664 | 673 | - | - | - | - | - | - |
| 2021 | Oct | 4,278 | 4,273 | 4,282 | - | - | - | - | - | - |
| 2021 | Nov | 10,227 | 10,222 | 10,231 | - | - | - | - | - | - |
| 2021 | Dec | 7,887 | 7,882 | 7,892 | - | - | - | - | - | - |
| 2022 | Jan | 341 | 337 | 346 | - | - | - | - | - | - |
| 2022 | Feb | 76 | 71 | 81 | - | - | - | - | - | - |
| 2022 | Mar | 3,792 | 3,787 | 3,797 | - | - | - | - | - | - |
| 2022 | Apr | 3,444 | 3,439 | 3,449 | - | - | - | - | - | - |
| 2022 | May | 8,911 | 8,907 | 8,916 | - | - | - | - | - | - |
| 2022 | Jun | 10,352 | 10,347 | 10,357 | - | - | - | - | - | - |
| 2022 | Jul | 6,333 | 6,328 | 6,337 | - | - | - | - | - | - |
| 2022 | Aug | 13,651 | 13,647 | 13,656 | - | - | - | - | - | - |
| 2022 | Oct | 7,584 | 7,579 | 7,589 | - | - | - | - | - | - |
| 2022 | Dec | 38 | 33 | 43 | - | - | - | - | - | - |
| 2023 | Jan | - | - | - | - | - | - | - | - | - |
| 2023 | Feb | 1,867 | 1,864 | 1,870 | - | - | - | - | - | - |
| 2023 | Mar | 654 | 651 | 656 | 260 | 200 | 330 | - | - | - |
| 2023 | Apr | 3,968 | 3,963 | 3,973 | 540 | 470 | 620 | - | - | - |
| 2023 | May | 5,088 | 5,083 | 5,093 | 203 | 120 | 250 | - | - | - |
| 2023 | Jun | 7,515 | 7,511 | 7,520 | 470 | 300 | 520 | - | - | - |
| 2023 | Jul | 19,466 | 19,457 | 19,474 | 241 | 80 | 302 | - | - | - |
| 2023 | Aug | 7,562 | 7,552 | 7,572 | 220 | 140 | 265 | - | - | - |
| 2023 | Sep | 420 | 410 | 430 | 199 | 140 | 220 | - | - | - |
| 2023 | Oct | 14,891 | 14,882 | 14,900 | 260 | 220 | 300 | 19 | 1 | 48 |
| 2023 | Nov | - | - | - | - | - | - | - | - | - |
| 2023 | Dec | 3,874 | 3,869 | 3,880 | - | - | - | 96 | 22 | 187 |
| 2024 | Jan | - | - | - | - | - | - | - | - | - |
| 2024 | Feb | 279 | 274 | 285 | - | - | - | 147 | 43 | 282 |
| 2024 | Mar | 6,891 | 6,886 | 6,896 | - | - | - | - | - | - |
| 2024 | Apr | - | - | - | 470 | 390 | 550 | 301 | 112 | 555 |
| 2024 | May | 13,968 | 13,956 | 13,980 | 390 | 300 | 480 | 188 | 66 | 359 |
| 2024 | Jun | 13,875 | 13,868 | 13,882 | 726 | 500 | 888 | 198 | 64 | 361 |
| 2024 | Jul | - | - | - | 816 | 489 | 890 | 38 | 4 | 85 |
| 2024 | Aug | 13,782 | 13,778 | 13,786 | 310 | 240 | 390 | 117 | 33 | 229 |
| 2024 | Sep | 9,545 | 9,540 | 9,550 | 420 | 350 | 490 | - | - | - |
| 2024 | Oct | 6,984 | 6,979 | 6,989 | 60 | 40 | 80 | 10 | 0 | 31 |
| 2024 | Nov | 47 | 41 | 52 | - | - | - | 198 | 65 | 367 |
